# Associations Between Dietary Intakes of Omega-3 Fatty Acids, Blood Levels, and Pain Interference in People with Migraine: A Path Analysis of Randomized Trial Data

**DOI:** 10.3390/nu18010003

**Published:** 2025-12-19

**Authors:** Jinyoung Park, Zachary O. Kadro, Gilson D. Honvoh, Anthony F. Domeniciello, Christopher E. Ramsden, Keturah R. Faurot, Vanessa E. Miller

**Affiliations:** 1 Department of Psychology and Neuroscience, Duke University, Durham, NC 27708, USA; 2 Department of Family Medicine and Community Health, Oakland University William Beaumont School of Medicine, Rochester, MI 48309, USA; 3 Integrative Medicine, Corewell Health William Beaumont University Hospital, Royal Oak, MI 48073, USA; 4 Integrative Medicine Program, Fred Hutchinson Cancer Center, Seattle, WA 98109, USA; 5 Cancer Prevention Program, Fred Hutchinson Cancer Center, Seattle, WA 98109, USA; 6 Department of Medical Oncology, University of Washington School of Medicine, Seattle, WA 98195, USA; 7 College of Nursing, University of Tennessee, Knoxville, TN 37996, USA; 8 Lipid Peroxidation Unit, National Institute on Aging, National Institutes of Health, Baltimore, MD 21224, USA; anthony.domenichiello@nih.gov (A.F.D.);; 9 Department of Physical Medicine and Rehabilitation, University of North Carolina at Chapel Hill, Chapel Hill, NC 27599, USA; 10 Department of Obstetrics and Gynecology, University of North Carolina at Chapel Hill, Chapel Hill, NC 27599, USA

**Keywords:** omega-3 fatty acids, dietary intervention, migraine

## Abstract

**Background/Objectives:** Increasing evidence supports the hypothesis that dietary intervention can improve pain among individuals with headaches, including migraine, a highly prevalent condition that can be disabling. Non-pharmacologic treatments for migraine are particularly attractive. In this secondary analysis of 182 participants enrolled in a randomized controlled trial of a dietary intervention designed to increase omega-3 (*n*-3) compared with a control diet, we examined the effects of eicosapentaenoic acid (EPA) and docosahexaenoic acid (DHA), both thought to decrease inflammatory processes. **Methods:** Path models with two time points (baseline and 16 weeks after randomization), were used to test the relationships between exposures of *n*-3 blood levels and self-reported dietary intake on outcomes of pain interference using the PROMIS pain interference scale and the Headache Impact Test (HIT-6). Model building was based on our published conceptual model. **Results:** Good fit was demonstrated for both models (EPA model: CFI = 0.984, RMSEA = 0.039, and SRMR = 0.045; DHA model: CFI = 0.981, RMSEA = 0.040, and SRMR = 0.040). Both EPA and DHA in the blood at 16 weeks were associated with lower levels of pain interference, but the effect for EPA was stronger (B = −0.56, *p* < 0.001 for EPA, and B = −0.43, *p* = 0.057 for DHA). **Conclusions:** Our findings are consistent with an indirect pathway linking diet to pain interference through blood levels of EPA and DHA in migraine.

## 1. Introduction

Migraine is a disabling condition impacting approximately 10% of the population [1]. Migraine symptoms include a moderate-to-severe headache often accompanied by photophobia, phonophobia, and nausea or vomiting [2]. Despite improvements in acute and preventative pharmacologic treatments, individuals with migraine often have a continued impact on their lives [3] as captured in pain interference measures [4,5]. Pain interference extends the assessment of pain to measure the degree to which pain interferes with or impacts a person’s physical abilities and enjoyment of life [6]. It is a concept adjacent to pain-related quality of life and is predictive of response to the long-term success of pain management [7]. Pain interference is often measured in headache studies using instruments such as the Headache Impact Test [4,8]. Non-pharmacologic therapies like dietary interventions have the potential to impact pain and pain-related outcomes, such as pain interference [9,10]. One such intervention is a diet that increases omega-3 (*n*-3) with or without decreasing omega-6 (*n*-6) intakes.

Evidence suggests that increasing *n*-3 polyunsaturated fatty acids (*n*-3 PUFA: EPA and DHA) in the diet can lead to improvements in pain outcomes among individuals with headache [11,12,13]. Less clear is the interrelationship of dietary *n*-3 fatty acids, blood level *n*-3 fatty acids, and pain interference. Arachidonic acid (AA), an *n*-6 fatty acid, is used by the body to form signaling molecules called eicosanoids, e.g., prostaglandins and leukotrienes. EPA and DHA, *n*-3 fatty acids, form oxidized lipids (oxylipins) that play important roles in the resolution of inflammation [14,15]. Although dietary intakes of *n*-6 linoleic acid (LA) or AA have not been found to result in increased inflammatory markers by themselves, their intake may interfere with the anti-inflammatory effects of *n*-3 fatty acids as they compete for the same enzymes involved in elongation, desaturation and eicosanoid synthesis [16]. This is relevant to the migraine population because neuroinflammation and cytokines are now recognized as contributors to migraine pathogenesis. Furthermore, jugular venous samples during migraine attacks demonstrate elevated TNF-α and IL-6 levels compared to controls [17]. EPA and DHA also appear to inhibit platelet aggregation and lower oxidative stress, both of which may be associated with migraine pathogenesis [16,18,19]. Finally, EPA and DHA exert differential effects in various domains of human physiology, including cardiovascular function, sleep, pain, and structure and function of the brain and nervous system [20,21,22].

Here we present a secondary analysis of a completed 3-arm randomized, controlled, dietary intervention trial seeking to improve headache outcomes in individuals with migraine. In this study, a high *n*-3 diet, with or without reductions in *n*-6 LA intakes, was found to decrease headache frequency, intensity, and duration with additional benefit from reducing *n*-6 LA [12]. An examination of the secondary outcomes revealed beneficial effects of the high *n*-3 diets on perceived quality of life, migraine-associated disability, and pain interference [23]. An original aim of the study was to examine pathways linking diet assignment to dietary intakes to *n*-3 biomarkers to pain outcomes, including pain interference (Headache Impact Test) and duration as documented in our published protocol [24]. For this analysis, we chose a priori to include a second measured pain interference variable (PROMIS Pain Interference 4a) in addition to the Headache Impact Test, and we did not include headache hours per day. We preserved the original focus on pathways of mediation from randomized diet assignment to dietary intakes of *n*-3 to *n*-3 blood levels to pain interference. Also, a priori, we decided to focus on *n*-3 PUFA, combining the two active intervention diets to ensure an adequate sample size to run the structural equation models. We hypothesized that the association between diet assignment and pain interference would be mediated by dietary *n*-3 intakes, *n*-3 blood levels, and *n*-3 metabolites.

## 2. Materials and Methods

### 2.1. Design

This research is secondary data analysis of individuals enrolled in a 16-week randomized controlled dietary intervention trial for migraine. For complete details of the study protocol, please see our previously published protocol manuscript [24]. Briefly, study participants with episodic or chronic migraine who met inclusion criteria were randomized to either one of the intervention diets (high *n*-3 and average *n*-6 or high *n*-3 and reduced *n*-6) or the control diet (average intakes of *n*-3 and *n*-6 in the United States). Randomization, under the exclusive control of the programmer, was concealed through a computerized system that utilized randomized blocks. All study personnel except the dietitians were masked to treatment allocation as were healthcare providers. Participants, of necessity, were not masked, but did not know the study hypotheses—they were told that the trial would test three dietary interventions for preventing migraine.

Participants met with a study dietitian at randomization and every 2–3 weeks during the intervention. During this time, they received enough food for about 2/3 of their caloric intake needs. Study foods included high *n*-3 fish for the intervention groups and low *n*-3 fish along with lean poultry for the control group. All participants received study oils which included a combination of olive and macadamia nut oil (for the low *n*-6 group) or a combination of olive and corn oil (for the average *n*-6 groups). A detailed description of the study diets is available [25]. Variables for this analysis were collected at randomization and at the end of the active intervention period (Week 16).

### 2.2. Participants

Participants who met International Headache Society criteria for episodic or chronic migraine [26] were recruited from academic, private practice clinics, and the surrounding community. All participants were under the care of a physician for migraine treatment and could continue usual care as prescribed, but they were asked to refrain from adding therapies not specifically recommended by their physicians. Participants were permitted to take migraine prevention and abortive medications and recorded the dose of abortive medications in a daily online diary.

Adults with 5–20 migraines per month with a history of migraines for two or more years who were willing to be randomized to intervention diets were included in the trial. Individuals were excluded if they: (1) were pregnant or intending to become pregnant; (2) had changed hormone therapy in the previous 6 months; (3) had an active serious psychological or physical illness; (4) had a recent head injury; (5) had cognitive impairment preventing consent; (6) required management in a pain clinic; (7) had a serious food allergy or aversion to fish; (8) were currently on a weight loss diet or investigational study; or (9) had been taking fish oil supplements regularly.

Ethics approval was provided by the Human Research Ethics Committee of the University of North Carolina (IRB# 13-3284), and all participants provided written informed consent. The study was registered in ClinicalTrials.gov (NCT02012790).

### 2.3. Measures

The Headache Impact Test (HIT-6) is a self-reported headache-specific pain interference measure. It is designed to assess the impact of headaches on daily function over the past 4 weeks. The HIT-6 includes questions about the frequency of severe headaches, the association of the headache with fatigue, irritability, and lower concentration, and the perceived role of headaches in limiting usual daily activities, including social functioning [27]. As such, it covers both physical and social domains of pain interference. The HIT-6 has been validated in multiple headache populations, including chronic and episodic migraine [28,29]. Scores on the HIT-6 range from 36 to 78 with scores 60 or greater indicating a severe impact of headaches on quality of life. A minimal clinically important difference in the HIT-6 appears to depend on the population. In a primary care headache population, a difference of 1.5 points was consistent with an assessment of “somewhat better” headaches [30]. In a population with chronic migraine recruited into a pharmacologic treatment trial, a difference of 6 points was considered important [31].

The Patient-Reported Outcomes Measurement Information System (PROMIS) Pain Interference instrument uses self-report to measure how pain interferes with a person’s life and hinders their engagement in the following domains: social, cognitive, emotional, physical, and recreation [32]. There are multiple short-form versions of the instrument; the current study used the PROMIS Adult Short Form v1.0—Pain Interference 4a version [33]. The questionnaire asks people to respond to items over a 7-day recall period and mark the box that most reflects their experience. The four questions are (1) How much did pain interfere with your day-to-day activities? (2) How much did pain interfere with work around the home? (3) How much did pain interfere with your ability to participate in social activities? (4) How much did pain interfere with your household chores? Responders can mark one box for each question and the options include: not at all; a little bit; somewhat; quite a bit; or very much. The pain interference measure has been tested in clinical pain populations and found to be reliable and responsive to change. Meaningful differences in the t-scores rheumatoid arthritis populations were estimated to be between 5 and 7 points [34].

These two instruments were measured variables that contributed to the latent pain interference variable that was used in the structural equation model. Other variables in the model included the assigned diet (either one of the intervention diets or the control diet), dietary intake of *n*-3 fatty acids at baseline and during the intervention, and *n*-3 levels in white blood cells and plasma at baseline and at Week 16. Dietary intake was collected and analyzed with two unannounced 24 h food recalls in the pre-intervention period and after 10 weeks on the diet using a standardized protocol with the Nutrition Data System for Research developed by the Nutrition Coordinating Center, University of Minnesota, Minneapolis, MN. Fasting blood samples were drawn for biochemical analysis. Samples were aliquoted immediately and stored at −80 °C until processing with liquid chromatography [24].

### 2.4. Data Analyses

The analysis plan, including the structural model, was outlined in the protocol paper. a priori modifications to the protocol-specified model involved including a second pain interference measure, collapsing the two intervention diets groups into one, and removing the *n*-3 metabolites, which showed very low correlations with the other blood *n*-3 indicators. Because they did not meaningfully contribute to the underlying construct, the metabolites were excluded from the final model. Analyses were conducted using R version 4.4.1 (R Foundation for Statistical Computing, Vienna, Austria). Univariate analyses were generated to summarize the data. All analyses utilized natural log transformations of both dietary intakes and blood measurements of EPA and DHA to normalize distributions.

**Figure 1:**
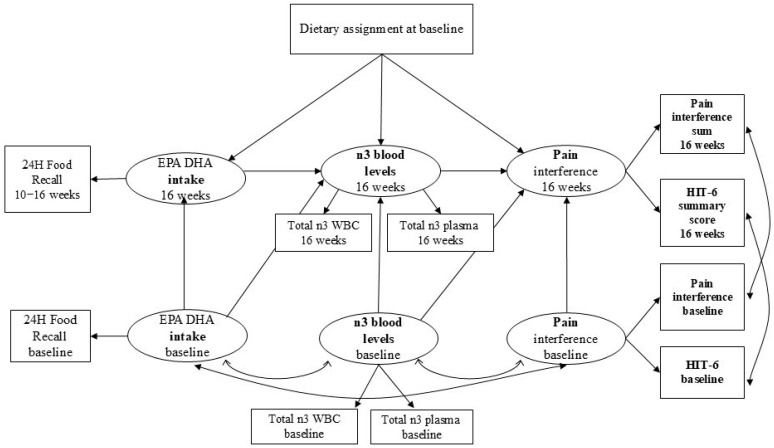
Conceptual model of the relationship between dietary intake, blood levels, and pain interference. Observed variables are depicted by rectangles. These include randomized diet assignment and the following variables measured at baseline and 16 weeks: (a) 24 h food intake; (b) *n*-3 fatty acids in white blood cells; (c) *n*-3 fatty acid levels in plasma; (d) PROMIS pain interference 4a; and (e) the HIT-6 score. Latent variables are depicted by ellipses. They include: (a) *n*-3 intakes at baseline and 16 weeks; (b) *n*-3 blood levels at baseline and 16 weeks; and pain interference at baseline and 16 weeks. Double-headed arrows represent an expected correlation between variables. The model hypothesizes that the effect of diet assignment on pain interference is mediated by EPA or DHA intakes and EPA or DHA blood levels. Abbreviations: 24H = Twenty-four hour; *n*3 = omega-3 fatty acid; EPA = eicosapentaenoic acid; DHA = docosahexaenoic acid; WBC = white blood cell; HIT-6 = Headache Impact Test.

The structural equation model (SEM) is used to test a set of hypothetical causal relationships between inter-related variables by examining the fit of the model-implied variance-covariance matrix to that of the observed data. We developed separate SEMs for EPA and DHA, respectively, to examine their distinct effects on outcomes. Latent variables were created to represent EPA (or DHA) intake, EPA (or DHA) blood levels, and the outcome of interest—headache-specific and general pain interference. A confirmatory factor analysis was used to assess the fit of the hypothesized measurement model. Measurement models used the HIT-6 and PROMIS pain interference measures to form the latent outcome variable. A log-transformed summary variable of EPA (DHA) intake was used to form a single indicator latent EPA (DHA) intake variable. The log-transformed EPA (DHA) levels in white blood cells (WBC) and plasma were used to create latent blood EPA (DHA) variables. Diet assignment was entered as an observed variable. Our conceptual model depicts the proposed relationships among the variables (Figure 1).

Models were evaluated first by assessing parameter estimates and model fit indices with established guidelines for good model fit, including absolute and incremental fit indices: (1) the root mean square error of approximation (RMSEA); (2) comparative fit index (CFI); (3) Tucker–Lewis index (TLI); and (4) the standardized root mean square residual (SRMR). Established values for model fitness were CFI/TLI values ≥ 0.95, RMSEA < 0.07, and SRMR < 0.09 [35]. We used full information maximum likelihood methods to account for missing data and to address non-normality of variable distributions. Standardized parameter estimates were used to allow for comparisons between measures assessing using different scales. Exploratory changes were made to improve model fit. EPA in the plasma at the baseline was set to have zero variance due to the negative variance issues.

Mediation analysis was performed to calculate the direct, indirect, and total effects of the diet assignment. Mediating variables were dietary intakes of EPA (or DHA) as measured through 24 h food recalls prior to the 16-week time point (at approximately Week 13) and blood levels (EPA or DHA levels in plasma and WBCs) at 16 weeks. The direct effect was defined as the effect of diet assignment on the outcomes, excluding the effect gained through the mediators. The indirect effect was defined as the mediational effect, representing the amount of intervention effect accounted for by mediators (Figure 1). The total effect of the diet assignment is calculated by summing the direct and indirect effects.

To control for the carry-over effects and model the change from the baseline, both mediators and outcomes at the baseline time point were included in the model. The following simplified equation illustrates the model structure:

$$Y_{post}=\beta_{pre}^{Y}Y_{pre\;}+\;\beta_{post}^{M}M_{post}+\beta_{pre}^{M}M_{pre}+\beta_{pre}^{E}E_{pre}+\varepsilon$$

where $Y_{pre}$ and $Y_{post}$ are the outcomes (pain interference) at baseline and week 16, respectively, $M_{post}$ and $M_{pre}$ are the mediators (EPA dietary intakes and blood levels or DHA dietary intakes and blood levels) at baseline and week 16, $E_{pre}$ is the diet assignment, $\varepsilon \sim Ν\left(0,\sigma^{2}\right)$, and $\beta_{\cdot }^{\cdot }$ are the coefficients of the corresponding variables and time points.

Because this study was a randomized controlled trial, diet assignment can be assumed to be independent of baseline participant characteristics; therefore, for the primary analysis, the SEMs did not adjust for additional baseline covariates. However, the mediation analysis still relies on the standard—but untestable—assumption that no important unmeasured confounders exist for the relationship between blood EPA/DHA and pain interference that are themselves unaffected by diet assignment. Violations of this assumption would bias the estimated direct effect, which may, therefore, reflect any residual influence of such confounders. A sensitivity analysis examined a model controlling for measured variables we felt would be most likely to confound the association between blood EPA/DHA and pain interference: baseline age, BMI, botulinum toxin use, and depression.

## 3. Results

### 3.1. Description of Sample

A total of 182 participants (M 38.3 years, SD 12.0 years) were randomized at the beginning of follow-up. Table 1 provides the descriptive statistics for the baseline variables of our analytic sample. The majority (75.8%, *n* = 138) of the participants were White, 18.1% were Black/African American and 6.0% were Asian/Native Americans/Pacific Islanders. Most were women (88.5%), married or living with a partner (66.3%), and had at least an undergraduate degree (92.8%). Additional baseline characteristics can be found in the primary outcomes paper [12].

The variables included from our theoretical causal pathway are presented in Table 2. Average changes between baseline and week 16 in *n*-3 intakes from food were 0.2 g/day for EPA and 0.6 g/day for DHA. These average changes between baseline and week 16 resulted in an increase in 1.6 (log ng/mL) in WBC and 8.4 (log ng/mL) in plasma for EPA, and to 2.6 in WBC and 19.2 in plasma for DHA.

We performed paired *t*-tests to assess pre-post changes for all variables in Table 2. All variables showed a statistically significant change between baseline and Week 16 (*p* < 0.01). Pain interference measures showed improvement from baseline to week 16 with a decrease in pain interference z-score of 4.6 and a decrease in HIT-6 of 4.1.

### 3.2. Model Results

The model was built to test the theoretical causal relationship between EPA (or DHA) intake and the outcomes of interest. As is commonly observed in nutrition studies, the diet assignment served as an instrumental variable due to the variability in actual intake. The mediation model reflected the dynamics, where the diet assignment affected the outcomes only through the actual intakes of EPA (or DHA), followed by the intakes predicting the blood level of EPA (or DHA), and finally, the blood level predicting the outcomes (Figure 1).

**Figure 2:**
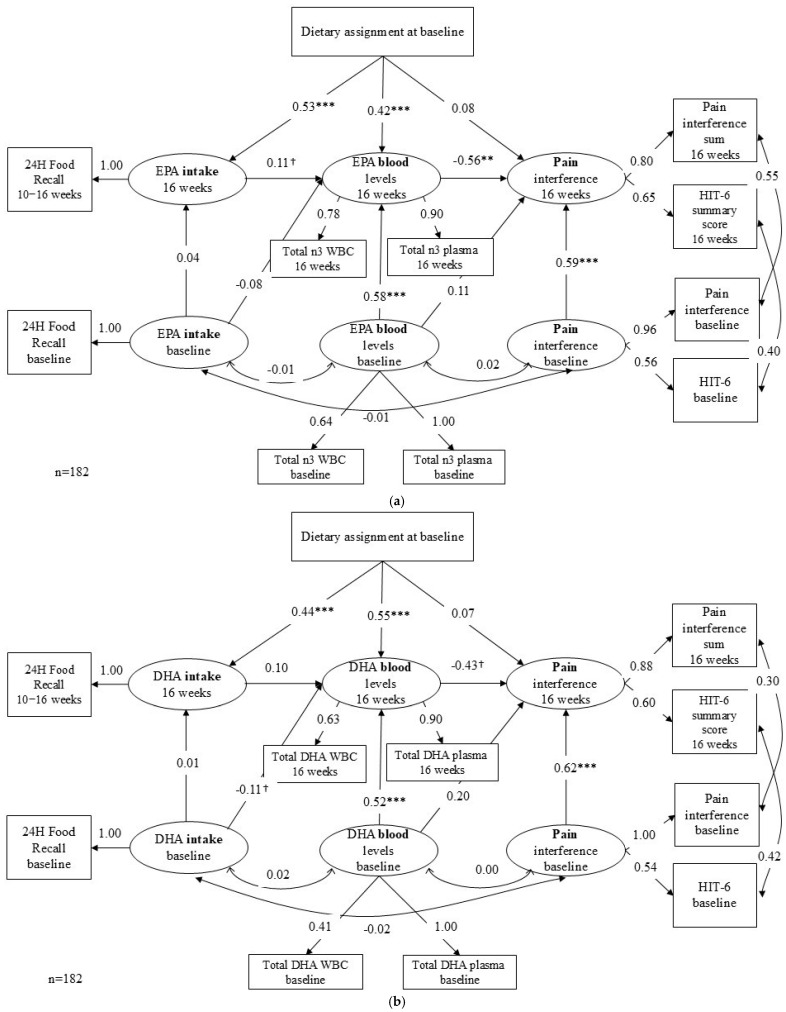
(**a**) Final EPA Model. (**b**) Final DHA Model. Note. Rectangles represent observed variables, and ellipses represent latent variables. Double-headed arrows represent an expected correlation between variables. Variances, disturbances, residuals, and covariances among residuals are omitted for clarity. Values shown are standardized path coefficients. ^†^ *p* < 0.10, ** *p* < 0.01, *** *p* < 0.001. Abbreviations: 24H = Twenty-four hour; *n*3 = omega-3 fatty acid; EPA = eicosapentaenoic acid; DHA = docosahexaenoic acid; WBC = white blood cell; HIT-6 = Headache Impact Test.

Overall, both the EPA (Figure 2a) and the DHA (Figure 2b) models had satisfactory fit indices. In the EPA model, Robust chi-square χ^2^_(df=33)_ = 41.01, *p*-value = 0.159, Robust CFI = 0.99, Robust RMSEA = 0.039, and SRMR = 0.045. For the DHA model, Robust chi-sqare χ^2^_(df=33)_ = 40.79, *p*-value = 0.165, Robust CFI = 0.981, Robust RMSEA = 0.040, and SRMR = 0.040 (Supplemental Table S1).

#### 3.2.1. Path 1 & 2: Diet Assignment to Food Intake to Blood *n*-3 Level

As expected, the diet assignment was significantly linked to recorded food intake (B = 0.53, *p* < 0.001 for EPA, B = 0.44, *p* < 0.001 for DHA) and also to blood EPA and DHA levels (B = 0.42, *p* < 0.001 for EPA, B = 0.55, *p* < 0.001 = for DHA) controlling for baseline intake and baseline blood levels. Unexpectedly, increased *n*-3 intake showed only a weak association with blood EPA and DHA levels after controlling for diet assignment and baseline blood levels (EPA: B = 0.11, *p* = 0.07; DHA: B = 0.10, *p* = 0.12).

#### 3.2.2. Path 3: Blood *n*-3 to Pain Interference

EPA and DHA showed a slightly different pattern in predicting outcomes. Both EPA and DHA in the blood at week 16 were associated with lower levels of pain interference, but the effect for EPA was stronger (B = −0.56, *p* < 0.001 = for EPA, B = −0.43, *p* = 0.057 for DHA).

#### 3.2.3. Indirect Effect and Direct Effect

The mediation effect of diet assignment on reduced pain through dietary intake and blood *n*-3 levels was not statistically significant for either EPA (B = −0.03, *p* = 0.07) or DHA (B = −0.02, *p* = 0.15). In contrast, the indirect effect of diet assignment on reduced pain interference through blood *n*-3 levels alone was significant for EPA (B = −0.23, *p* = 0.008), but not for DHA (B = −0.23, *p* = 0.062). These standardized effects (−0.23) correspond roughly to a 2.3-point decrease on the HIT-6, and a 3.2 decrease in PROMIS pain interference t-score. None of the direct effects were statistically significant (EPA: B = 0.08, *p* = 0.441; DHA: B = 0.07, *p* = 0.566).

Overall, these findings suggest that the effect of diet assignment on pain reduction is fully mediated by increases in blood *n*-3 levels, particularly for the EPA model, rather than by changes in dietary intake.

#### 3.2.4. Sensitivity Analysis Results

Including the four potential confounding variables had little effect on the model variable interpretations. The indirect effect of diet assignment on reduced pain interference through EPA and DHA was slightly stronger (−0.27) with smaller *p* values (0.006 for EPA and 0.011 for DHA). The associations between EPA or DHA blood levels and pain interference were also stronger (Supplemental Table S2).

## 4. Discussion

### 4.1. Key Findings

We present the results of structural equation modeling to assess the validity of a conceptual model demonstrating improvement in headache pain interference measures (PROMIS pain interference and headache impact) in a randomized trial. In this secondary analysis of the randomized trial, we tested our conceptual model of circulating levels of EPA and DHA, along with self-reported measures of food intake and the randomization to either a control diet or a diet high in EPA and DHA. We controlled for the same baseline measures of pain interference that were assessed after 16 weeks of a dietary intervention. The structural equation model allowed for testing of direct and indirect effects of EPA and DHA on pain interference measures. As we expected, we found a moderately strong effect of the baseline headache pain interference measures on pain interference measured at 16 weeks. However, although we hypothesized that the effect of diet assignment on pain interference would be mediated by both dietary *n*-3 intakes and *n*-3 blood levels, we did not find that dietary intakes were an important mediator, nor did we find a direct effect of diet assignment. Instead, our findings indicate that the data are consistent with an indirect pathway from diet to pain through EPA or DHA in blood. Our primary mediation models did not adjust for potential baseline covariates that might confound the relationship between blood EPA or DHA and pain interference. As a result, the indirect effects should be interpreted under the assumption of no important unmeasured mediator–outcome confounding. However, a sensitivity analysis controlling for age, BMI, botulinum toxin use, and depression resulted in no appreciable model changes.

The association between *n*-3 dietary intake and *n*-3 blood levels was weak, suggesting possible error in our dietary assessment or the influence of unmeasured factors. The most reliable measure of dietary intake requires multiple unannounced food recalls, ideally more than three [36,37]. Because our study included at most two recalls per participant, we may not have captured intakes adequately [25,38]. Under optimal conditions, correlations between the average of four 24 h food recalls and plasma fatty acid levels were only about 0.4–0.6 [38]. Moreover, the recall collected after 10 weeks on the diet may have been affected by social desirability bias, as participants knew that they were expected to consume provided foods and adhere to the protocol. In a previous validation study, adding measures of social desirability to regression calibration equations helped reduce bias associated with self-reported dietary intakes by accounting for participants’ tendency to over- or under-report certain foods [39]. Unmeasured factors may also explain the weak association, such as genetic polymorphisms affecting *n*-3 fatty acid metabolism that influence absorption [40].

### 4.2. Previous Studies

Our results align with previous findings on the effects of EPA and DHA on headache and pain in this trial’s migraine population. In Ramsden et al. (2021), higher EPA and DHA were found to be associated with significant reductions in headache hours per day and headache days per month [12]. While also showing consistent directionality in reducing pain, the associations with the HIT-6 were not statistically significant. However, our path analysis uncovered additional patterns, including a possible mediating effect of the blood fatty acids. In a previous study (*n* = 67) comparing the H3L6 (high *n*-3, low *n*-6) diet to a diet low in *n*-6 PUFA, the diet increasing *n*-3 was found to have significant improvements in both headache days per month (*p* = 0.02), the HIT-6 (*p* < 0.001), and erythrocyte EPA and DHA (*p* < 0.001) [11].

Recent studies have examined the effect of increasing *n*-3 through fish oil supplements on headache outcomes, including disability, quality of life, and pain interference. In a small study (*n* = 70) comparing 1800 mg EPA in ethyl ester form to a placebo over 12 weeks, the Migraine Disability Assessment test improved by a mean (M) of 13.1 (SD 16.2) in the EPA group compared with a worsening of M 2.6 (SD 20.2) in the control group (*p* = 0.001). Similarly, the authors reported a statistically significant change in the Migraine-Specific Quality of Life Score (*p* = 0.007) between the groups [41]. The authors also reported significant differences between groups in headache frequency, severity, and abortive medication use. The study did not report blood levels of EPA. Another small study (*n* = 60) examined the effects of 2g EPA vs. placebo in chronic migraine over 8 weeks, reporting significant improvements in the HIT-6 (time by group interaction *p* = 0.026) and headache frequency (*p* < 0.001) but not headache severity. EPA blood levels are not reported. In another small study (*n* = 60) of *n*-3 supplementation (1.5 g of EPA + DHA) in patients with chronic migraine taking amitriptyline, the authors reported significant reductions in headache frequency (*p* = 0.038) but did not report pain interference or fatty acid blood levels [42]. Limitations of these studies were the absence of EPA and DHA blood level measurements and small sample sizes.

### 4.3. Study Limitations and Strengths

A strength of this study is the multiple ways that EPA and DHA were assessed in study participants, including results from self-reported intake, observed levels from blood samples, and the effect of randomization to a diet high in EPA and DHA compared with a control diet. Another strength is our study’s contribution to the pain interference literature in the migraine population; pain interference has been measured in other migraine studies, but infrequently in nutrition studies related to migraine. For example, Rebecca Wells et al., using a mindfulness-based stress reduction (MBSR) intervention for migraine, showed a positive impact on the Migraine Specific Quality of Life (MSQv2.1) and HIT-6 measures, though not a significant impact on pain frequency as measured by the number of migraine days per month [43]. There is an important distinction between pain and pain interference because pain interference captures the emotional and functional effects of migraine more than pain frequency, e.g., number of headache days per month, which is currently considered the gold standard for pain measurement in migraine studies. Pain interference attempts to assess how pain impacts people’s functioning in everyday life.

Important limitations exist for this research. The study sample included mostly women (>88%), and >90% had at least a college education, limiting the generalizability of the findings. The demographics of the study sample reflect ongoing challenges in pain research to broadly reflect the general population [44]. Also, even though the sample size was deemed to be adequate to demonstrate differences between the intervention groups and a control group with average U.S. intakes of *n*-3 and *n*-6, the study was not powered for mediation analyses, particularly those that include not only dietary intakes and blood levels of EPA and DHA, but also EPA and DHA metabolites that may be implicated in pain pathways.

It should be noted that in this manuscript, we report circulating concentrations of fatty acids that do not necessarily reflect incorporation of fatty acids into tissue. Though some molecules in circulation may impact migraine [45,46], it is not known whether fatty acids would have similar effects. Therefore, it is possible that the effect of EPA and DHA in the models presented here would be different if different tissues were analyzed/included.

### 4.4. Next Steps and Gaps

This study demonstrates the importance of considering biomarkers in addition to dietary intake when assessing EPA and DHA. Many previous studies did not measure blood levels of DHA and EPA nor their metabolites. The next steps in this field of investigation studying the effects of H3L6 diets compared to a control includes whether dietitian support instead of food provision is adequate for people to implement these dietary changes on their own. Another next step is to test the H3L6 hypothesis using a moderately low *n*-6 diet in conjunction with an *n*-3 fish oil supplement. It is unclear if increasing *n*-3 with a supplement will achieve similar results in both biochemical and clinical outcomes. More broadly, next steps include seeing if this type of dietary intervention focused on increasing *n*-3 and decreasing *n*-6 intakes can reduce pain in other types of chronic pain conditions.

## 5. Conclusions

In this study of individuals with chronic and episodic migraine, our findings were consistent with an indirect pathway from diet to pain through blood levels of EPA and DHA. These results provide preliminary support for the potential benefit of increasing *n*-3 fatty acids to reduce pain interference in people with migraine, though additional research is warranted.

## Figures and Tables

**Table 1:** Baseline characteristics of the study population.

Characteristic		Total	Intervention	Control
Age	Mean (SD)	38.3 (12.0)	39.1 (11.8)	36.9 (12.5)
BMI	Mean (SD)	29.2 (7.5)	29.0 (7.7)	29.5 (7.3)
**Marital status**				
Married or living with a partner	N (%)	119 (66.5)	76 (63.9)	43 (71.7)
Single or widowed or divorced/separated	N (%)	60 (33.5)	43 (36.1)	17 (28.3)
**Highest education level**				
High school or less	N (%)	13 (7.2)	9 (7.4)	4 (6.8)
Undergraduate degree	N (%)	109 (60.6)	72 (59.5)	37 (62.7)
Graduate degree	N (%)	58 (32.2)	40 (33.1)	18 (30.5)
**Income**				
$41K and more	N (%)	108 (61.0)	76 (65.0)	32 (53.3)
$40K and less	N (%)	69 (39.0)	41 (35.0)	28 (46.7)
**Race**				
White	N (%)	138 (75.8)	91 (74.6)	47 (78.3)
Black/African American	N (%)	33 (18.1)	22 (18.0)	11 (18.3)
Asian/Native American/Pacific Islander	N (%)	11 (6.0)	9 (7.4)	2 (3.3)
**Sex**				
Female	N (%)	161 (88.5)	109 (89.3)	52 (86.7)
Male	N (%)	21 (11.5)	13 (10.7)	8 (13.3)

**Table 2:** Variables in the path analysis.

Latent Variables	Observed Variables	N	Min	Max	Mean (SD)	Difference (W16 − W0)	*p*-Value
*n*-3 intake							
	EPA intake (g)					0.2	<0.01
	Baseline	179	0.0	0.4	0.0 (0.1)		
	Week 16	143	0.0	2.0	0.3 (0.3)		
	DHA intake (g)					0.6	<0.01
	Baseline	179	0.0	0.9	0.1 (0.1)		
	Week 16	143	0.0	3.2	0.7 (0.7)		
*n*-3 biomarkers							
Blood EPA							
	EPA *n*3 WBC level (log ng/mL)					1.6	<0.01
	Baseline	168	0.3	11.5	2.4 (1.9)		
	Week 16	135	0.5	17.7	4.0 (3.4)		
	EPA *n*3 Plasma level (log ng/mL)					8.4	<0.01
	Baseline	172	2.2	43.7	11.6 (6.3)		
	Week 16	125	2.9	63.2	19.9 (13.0)		
Blood DHA	DHA *n*3 WBC level (log ng/mL)					2.6	<0.01
	Baseline	168	1.02	40.4	6.5 (5.4)		
	Week 16	135	1.03	43.1	9.3 (6.8)		
	DHA *n*3 Plasma level (log ng/mL)					19.2	<0.01
	Baseline	172	12.0	109.1	35.4 (13.2)		
	Week 16	125	11.7	136.8	54.1 (25.8)		
Pain Interference							
	Hit-6 score (36–78)					−4.1	<0.01
	Baseline	182	40.0	78.0	62.7 (5.3)		
	Week 16	138	38.0	78.0	59.0 (7.0)		
	PROMIS pain interference t-score					−4.6	<0.01
	Baseline	182	41.6	75.6	59.0 (6.8)		
	Week 16	138	41.6	75.6	54.5 (8.1)		

Intakes are based on 24 h food intakes. PROMIS measures are t-scores with a population mean of 50 and an SD of 10. *p* values are based on paired *t*-tests.

## Data Availability

The data presented in this study are available on request from the corresponding author subject to a Data Use Agreement with the University of North Carolina at Chapel Hill. Available data include the R code needed to replicate the analyses.

## Supplementary Materials

The following supporting information can be downloaded at: https://www.mdpi.com/article/10.3390/nu18010003/s1, Table S1. EPA and DHA model estimates. Table S2. EPA and DHA model estimates controlling for age, BMI, Botox use, and depression.
