# Supplementary material for: Associations Between Dietary Intakes of Omega-3 Fatty Acids, Blood Levels, and Pain Interference in People with Migraine: A Path Analysis of Randomized Trial Data
Source: Nutrients. 2025 Dec 19;18(1):3. doi: 10.3390/nu18010003 (PMC12787831; doi:10.3390/nu18010003)
Supplement: Supplementary file 1 [file nutrients-18-00003-s001.zip › nutrients-3981706-supplementary.pdf]

Table S1. EPA and DHA model estimates.

	EPA Model		DHA Model	
	Estimate (95% CI)	p	Estimate (95% CI)	p
	Regression Slopes		Regression Slopes	
Omega-3 intake (week 16)				
Diet assignment	0.53 [0.46, 0.60]	$<$ 0.001	0.44 [0.29, 0.59]	$<$ 0.001
Omega-3 intake (baseline)	0.04 [-0.08, 0.17]	0.484	0.01 [-0.07, 0.10]	0.745
Omega-3 in blood (week 16)				
Diet assignment	0.42 [0.28, 0.57]	$<$ 0.001	0.55 [0.43, 0.66]	$<$ 0.001
Omega-3 in blood (baseline)	0.59 [0.39, 0.79]	$<$ 0.001	0.52 [0.39, 0.66]	0.001
Omega-3 intake (baseline)	−0.08 [-0.22, 0.05]	0.178	−0.11 [-0.22, -0.00]	0.059
Omega-3 intake (week 16)	0.11 [0.02, 0.21]	0.068	0.10 [-0.01, 0.21]	0.118
Pain (week 16)				
Diet assignment	0.08 [-0.13, 0.29]	0.441	0.07 [-0.18,0.33]	0.556
Pain (baseline)	0.59 [0.39, 0.79]	$<$ 0.001	0.62 [0.43, 0.81]	$<$ 0.001
Omega-3 in blood (baseline)	0.11 [-0.15, 0.37]	0.383	0.20 [-0.05, 0.44]	0.145
Omega-3 in blood (week 16)	−0.56 [-0.85, -0.26]	0.002	−0.43 [-0.77, -0.09]	0.057
	Constructed		Constructed	
Indirect effects				
Assignment → Omega-3 intake → blood → Pain	−0.03 [-0.07, 0.00]	0.072	−0.02 [-0.04, 0.01]	0.152
Assignment → Omega-3 in blood → Pain	−0.23 [-0.39, -0.08]	0.008	−0.23 [-0.43, -0.04]	0.062
Direct effects				
Assignment → Pain	0.08 [-0.13, 0.29]	0.441	0.07 [-0.18, 0.33]	0.566
Total effects				
Assignment → Pain	−0.18 [-0.34, -0.03]	0.029	−0.18 [-0.33, -0.02]	0.075
	Fit Indices		Fit Indices	
$\chi^2(\mathrm{df} = 33)$	35.51		34.67	
CFI	0.99		1.00	
TLI	0.98		0.99	
RMSEA	0.04		0.02	
Scaled χ^2(df)	41.01 (33)	0.159	40.79 (33)	0.165

Estimate = Standardzied regression weight

Table S2. EPA and DHA model estimates controlling for age, BMI, Botox use, and depression.

	EPA Model		DHA Model	
	Estimate (95% CI)	p	Estimate (95% CI)	p
	Regression Slopes		Regression Slopes	
Omega-3 intake (week 16)				
Diet assignment	0.51 [0.44, 0.59]	< 0.001	0.43 [0.28, 0.58]	< 0.001
Omega-3 intake (baseline)	0.06 [-0.06, 0.19]	0.305	0.03 [-0.07, 0.13]	0.616
Age	0.15 [0.05, 0.25]	0.021	0.07 [-0.11, 0.25]	0.423
BMI	−0.05 [-0.12, 0.02]	0.176	−0.01 [-0.20, 0.19]	0.943
Botox use	−0.01 [-0.07, 0.06]	0.864	0.02 [-0.02, 0.06]	0.404
Depression (baseline)	−0.06 [-0.19, 0.07]	0.365	0.01 [-0.14, 0.16]	0.885
Omega-3 in blood (week 16)				
Diet assignment	0.42 [0.28, 0.56]	< 0.001	0.54 [0.43, 0.66]	< 0.001
Omega-3 in blood (baseline)	0.57 [0.43, 0.71]	< 0.001	0.48 [0.32, 0.63]	0.003
Omega-3 intake (baseline)	−0.06 [-0.19, 0.07]	0.356	−0.07 [-0.15, 0.01]	0.104
Omega-3 intake (week 16)	0.09 [-0.02, 0.20]	0.166	0.07 [-0.05, 0.20]	0.289
Age	0.12 [-0.03, 0.26]	0.125	0.16 [0.03, 0.29]	0.025
BMI	−0.14 [-0.27, -0.00]	0.041	−0.12 [-0.27, 0.03]	0.097
Botox use	−0.02 [-0.13, 0.08]	0.649	−0.06 [-0.18, 0.06]	0.305
Depression (baseline)	0.00 [-0.12, 0.13]	0.975	0.10 [-0.02, 0.22]	0.100
Pain (week 16)				
Diet assignment	0.11 [-0.10, 0.33]	0.317	0.11 [-0.14,0.36]	0.395
Pain (baseline)	0.59 [0.39, 0.79]	< 0.001	0.62 [0.44, 0.80]	< 0.001
Omega-3 in blood (baseline)	0.15 [-0.10, 0.41]	0.228	0.21 [-0.03, 0.46]	0.101
Omega-3 in blood (week 16)	−0.64 [-0.96, -0.32]	< 0.001	−0.49 [-0.87, -0.11]	0.008
Age	0.11 [-0.11, 0.34]	0.228	−0.02 [-0.30, 0.35]	0.887
BMI	−0.20 [-0.37, -0.04]	0.048	−0.22 [-0.38, -0.06]	0.072
Botox use	0.05 [-0.18, 0.27]	0.700	0.04 [-0.27, 0.35]	0.809
Depression (baseline)	0.05 [-0.10, 0.21]	0.524	0.10 [-0.07, 0.26]	0.070
	Constructed		Constructed	
Indirect effects				
Assignment → Omega-3 intake → blood → Pain	−0.03 [-0.07, 0.01]	0.164	−0.02 [-0.05, 0.01]	0.268
Assignment → Omega-3 in blood → Pain	−0.27 [-0.44, -0.10]	0.006	−0.27 [-0.48, -0.05]	0.011
Direct effects				
Assignment → Pain	0.11 [-0.10, 0.33]	0.317	0.11 [-0.14, 0.36]	0.395
Total effects				
Assignment → Pain	−0.19 [-0.34, -0.03]	0.027	−0.17 [-0.36, -0.00]	0.032

Estimate = Standardzied regression weight
